# Supplementary material for: Deep Mutational Scanning of SARS-CoV-2 Receptor Binding Domain Reveals Constraints on Folding and ACE2 Binding
Source: Cell. 2020 Sep 3;182(5):1295–1310.e20. doi: 10.1016/j.cell.2020.08.012 (PMC7418704; doi:10.1016/j.cell.2020.08.012)
Supplement: Data S1. Interactive Heatmap of Mutational Effects on Expression and Binding, Related to Figure 3 [file mmc3.zip › Data_S1.html]

Instructions | SARS-CoV-2 RBD DMS


# SARS-CoV-2 RBD DMS

### Instructions

- Hover over cells with mouse to reveal additional information.
- Select site subsets using the drop down menu below the plots.
- Change which sites are displayed by brushing the zoom bar and dragging the brush.
- Clear the zoom bar by double clicking it.
- Structural visualizations of the data are available via `dms-view` here
- Raw data available on GitHub
